# Idiopathic Spinal Epidural Hematoma: A Near Miss of a Rare Entity

**DOI:** 10.7759/cureus.68939

**Published:** 2024-09-08

**Authors:** Chin Y Wells, Benjamin Heigle, Prabhneet Pannu, Stephen Pham, Ziad Ghneim, Neelakanta Dadi

**Affiliations:** 1 Internal Medicine, Unity Health-White County Medical Center, Searcy, USA; 2 Hematology/Oncology, Unity Health-White County Medical Center, Searcy, USA

**Keywords:** back pain, laminectomy, lower limb weakness, spinal epidural hematoma, surgical decompression

## Abstract

Spinal epidural hematomas (SEH) are rare, and cases with a spontaneous etiology are even more infrequent. Management of spontaneous SEH varies, with surgical or conservative approaches determined by the severity of deficits and symptom resolution. Adverse prognostic factors may include thoracic segment location, anticoagulation use, severe neurologic deficits at admission, sphincter dysfunction, and rapid progression. We report a patient with a sudden onset of bilateral lower limb weakness and reduced urinary output. Magnetic resonance imaging was conducted and indicated an epidural hematoma extending from T11 to L4. Surgical decompression and hematoma extraction were performed successfully resulting in the complete resolution of symptoms. This case underscores the importance of considering spontaneous SEH in patients lacking conventional risk factors, such as a history of trauma, when presenting with symptoms of bilateral lower limb weakness and decreased urine output. Depending on the severity of symptoms and the occurrence of spontaneous and rapid improvement, the patient may benefit from surgical intervention, which ameliorated the patient's symptoms in this case.

## Introduction

Spinal epidural hematomas (SEH) are rare. The literature has documented approximately 1,100 SEH cases from any cause since 2021, with a reported prevalence of SEH at around 0.1 per 100,000 [1]. Furthermore, SEH exhibits a male-to-female ratio of approximately 1.5:1 and demonstrates a bimodal distribution in age and spinal location [2]. Predisposing factors include trauma, pregnancy, coagulopathy, and spinal puncture. Management of SEH varies, with surgical or conservative approaches determined by the severity of deficits and whether symptom resolution occurs spontaneously. According to a large retrospective case-control study in 2022, prognostic factors may include thoracic segment location, anticoagulation use, severe neurologic deficits at admission defined by sphincter dysfunction, and rapid progression [3]. Computed tomography (CT) is often the initial imaging choice, as magnetic resonance imaging (MRI) is not commonly employed as a first-line practice in emergency departments.

## Case presentation

An 86-year-old Caucasian woman presented to the emergency department with sudden-onset back pain and leg weakness. Her medical history included breast cancer, bladder cancer, and colon cancer status-post ostomy, chronic pedal neuropathy secondary to chemotherapy, osteoarthritis, and transient ischemic attack (TIA). The patient reported being on clopidogrel for over a decade due to her history of TIA but otherwise denied taking other antiplatelet or anticoagulation medicines. The back pain woke her around 5:00 a.m., and she discovered she could no longer use her legs. She said she swung her legs out of bed with her arms because her legs were too weak. Then, she suddenly developed sharp lower back pain. The pain was described as the worst back pain she had ever experienced. The patient was ambulatory with a cane at baseline but could not bear any weight the morning of symptom onset. After calling emergency services for help, she was sent to the emergency department for evaluation. There was no history of recent trauma or illness. She reported a remote history of lumbar spine surgery approximately 20 years ago and said that no hardware was implanted. She denied a history of acupuncture and other manipulations of her spine. She denied urinary incontinence, vomiting, chest pain, dysuria, cough, dyspnea, sore throat, and abdominal pain. She did endorse some nausea and recent decreased urinary output. She was unsure when her last positron emission tomography (PET) scan was; however, she was told that her cancer was still in remission. She has a history of extensive abdominal surgeries, including colon resection with ostomy, hernia repair, hysterectomy, and bladder surgery. She was a former smoker with approximately 25 pack-years; however, she has not smoked in 30 years. She does not drink alcohol or use drugs.

During admission to our emergency department, her body temperature was 36.5°C, her respiratory rate was 15 per minute, her pulse rate was 78 beats per minute (bpm), her blood pressure was 185/68 mm Hg, and her oxygen saturation was 96% on room air. The physical examinations of the head, neck, chest, and heart yielded normal findings. A colostomy was noted in her left abdomen. In both lower limbs, her muscle strength was 2 out of 5 [4]. The deep tendon reflex was normal (2+) in bilateral upper extremities but decreased (1+) in the lower limbs bilaterally. Proprioception on the left was impaired, and there was chronic loss of light touch to the feet bilaterally. No edema or wound was present over the extremities or spine. Tenderness over the lumbar spinous processes was identified. Laboratory workup demonstrated a potassium of 2.8 mmol/L, which was repleted in the emergency department. Otherwise, there were no significant findings. A CT angiogram of the aorta was ordered that was negative for aortic dissection, but it did reveal a distended gallbladder with pericholecystic fat stranding. No suspicious osseous lesions were noted. A follow-up abdominal ultrasound was ordered, revealing a focal mild wall thickening and pericholecystic fluid of the gallbladder fundus. A general surgeon was consulted, and a hepatobiliary iminodiacetic acid (HIDA) scan was recommended. The patient was then admitted for further workup.

**Figure 1:**
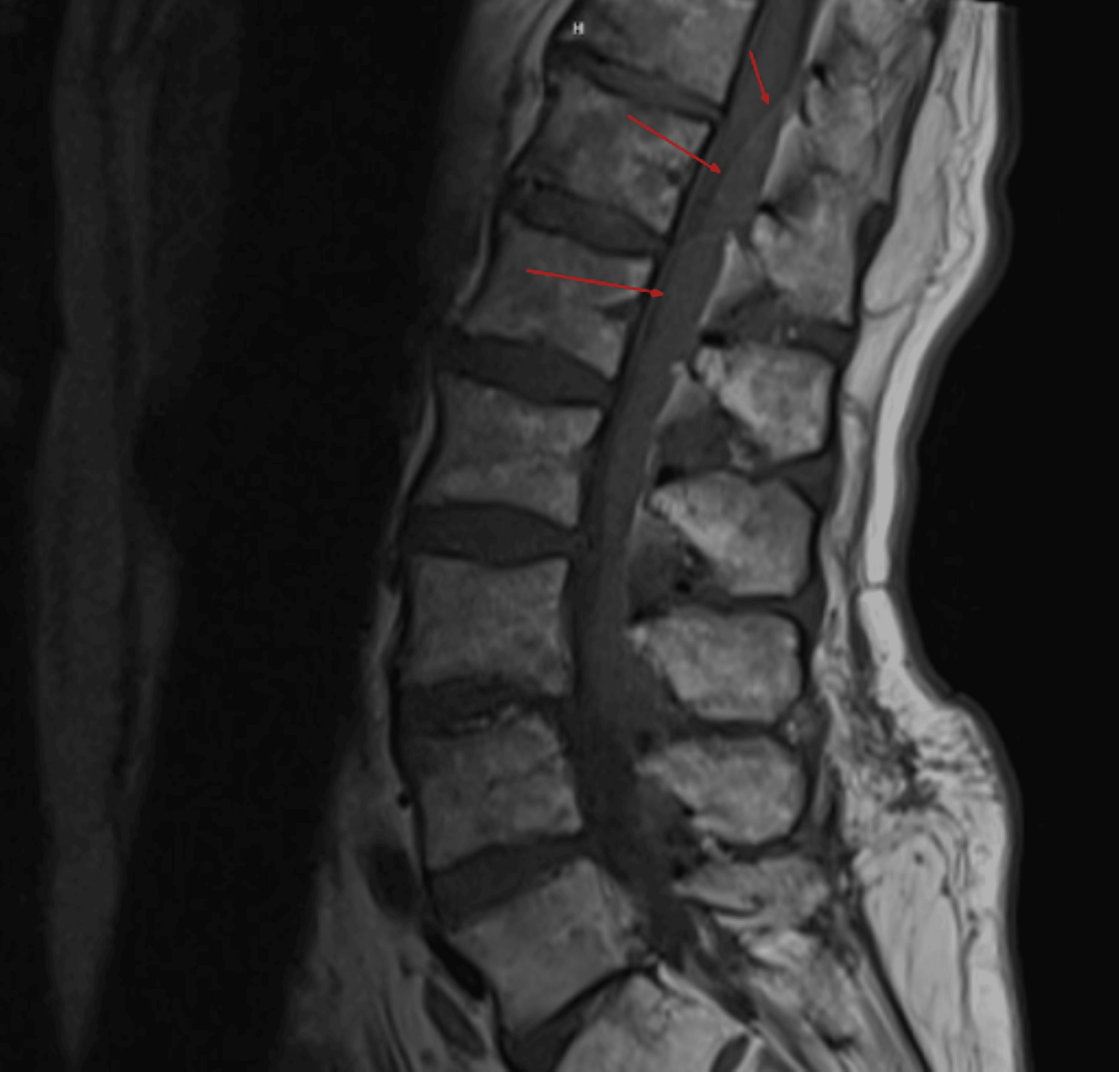
T1 sagittal MRI demonstrates a delineated hyperintensity within the spinal canal displacing the cord anteriorly. MRI: magnetic resonance imaging

**Figure 2:**
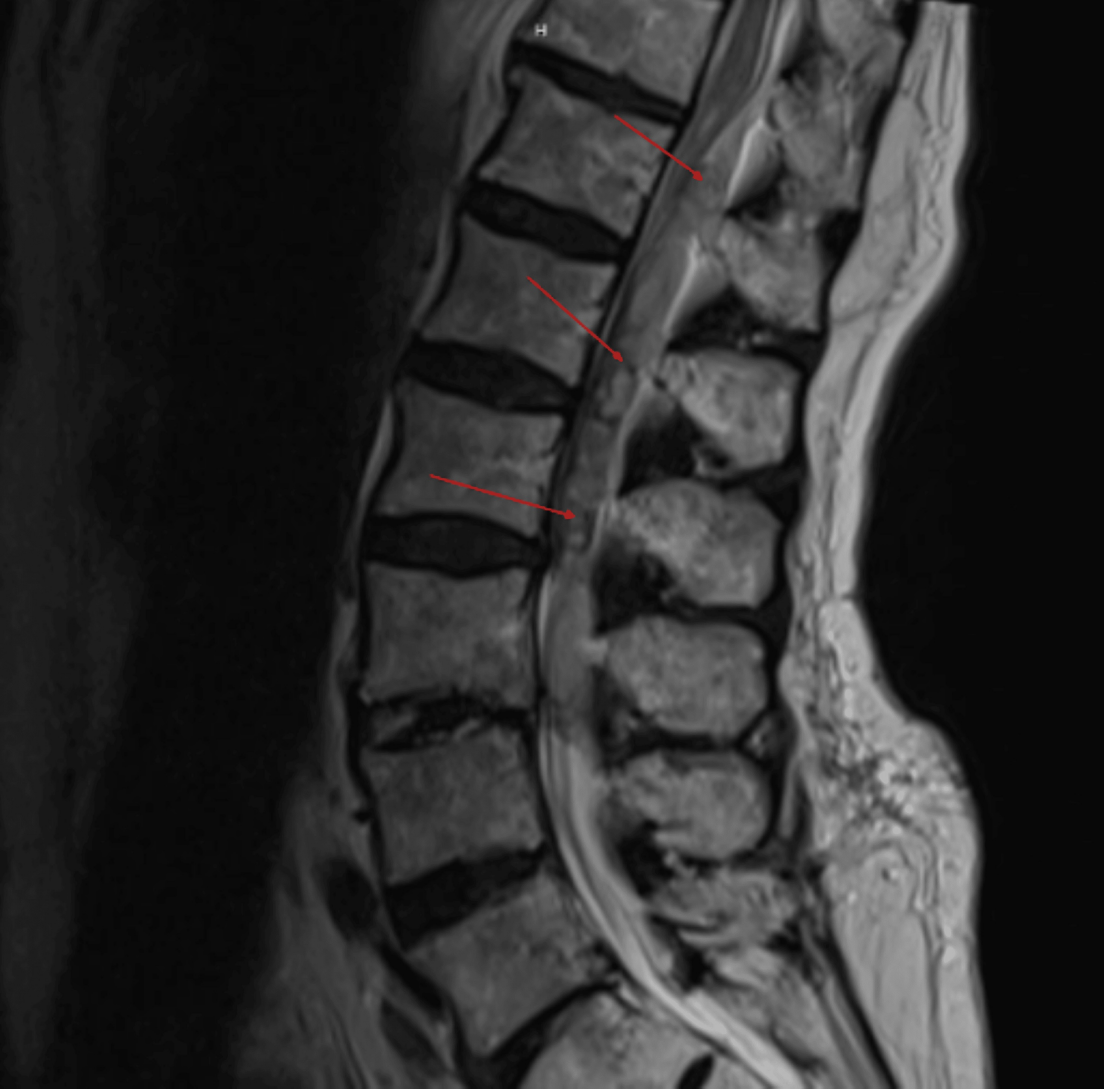
T2 sagittal MRI demonstrates hyperintensity with hypointense foci throughout. MRI: magnetic resonance imaging

**Figure 3:**
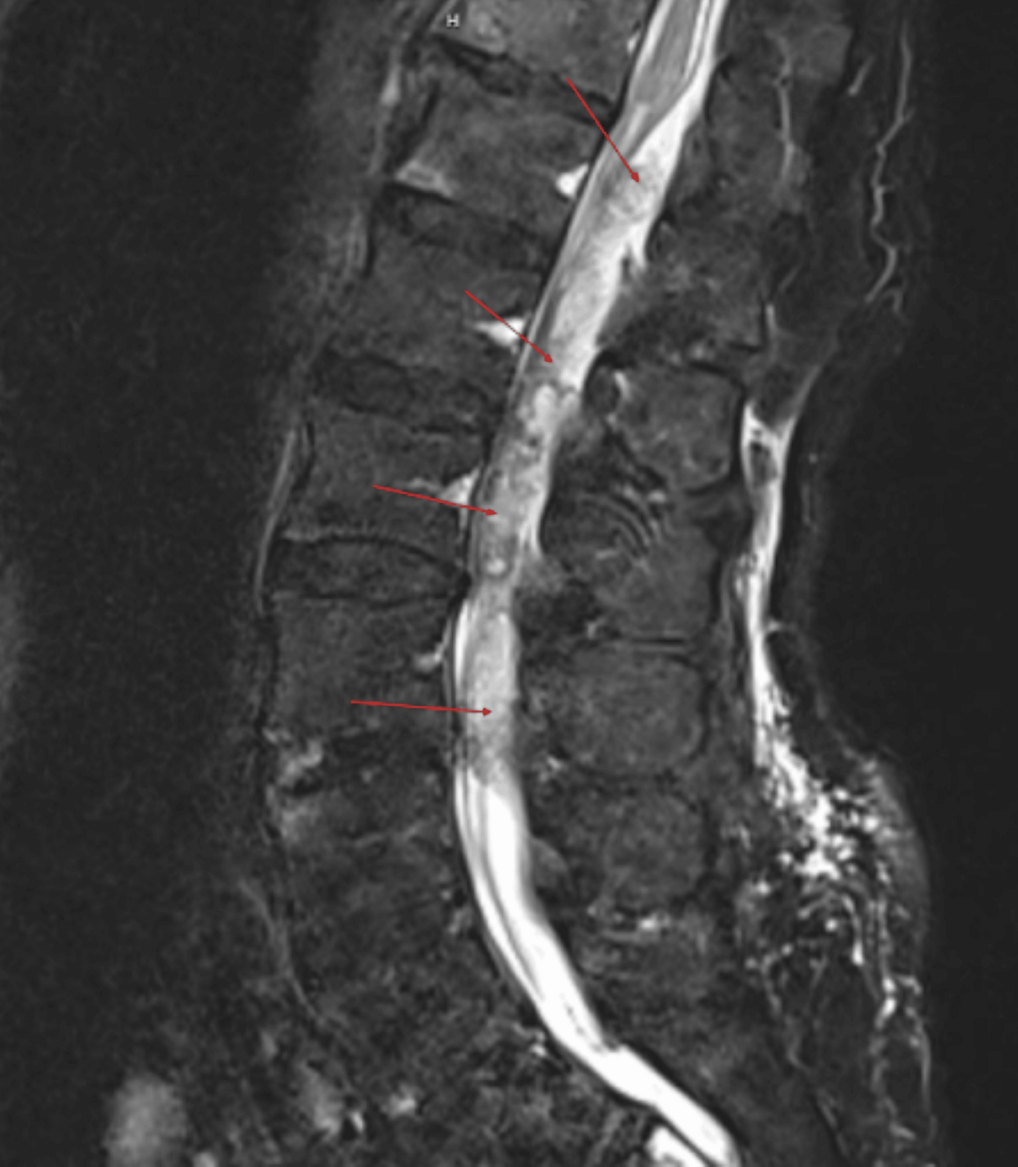
T2 fat suppression sequence again demonstrates a delineated hyperintensity within the spinal canal and hypointense foci throughout.

**Figure 4:**
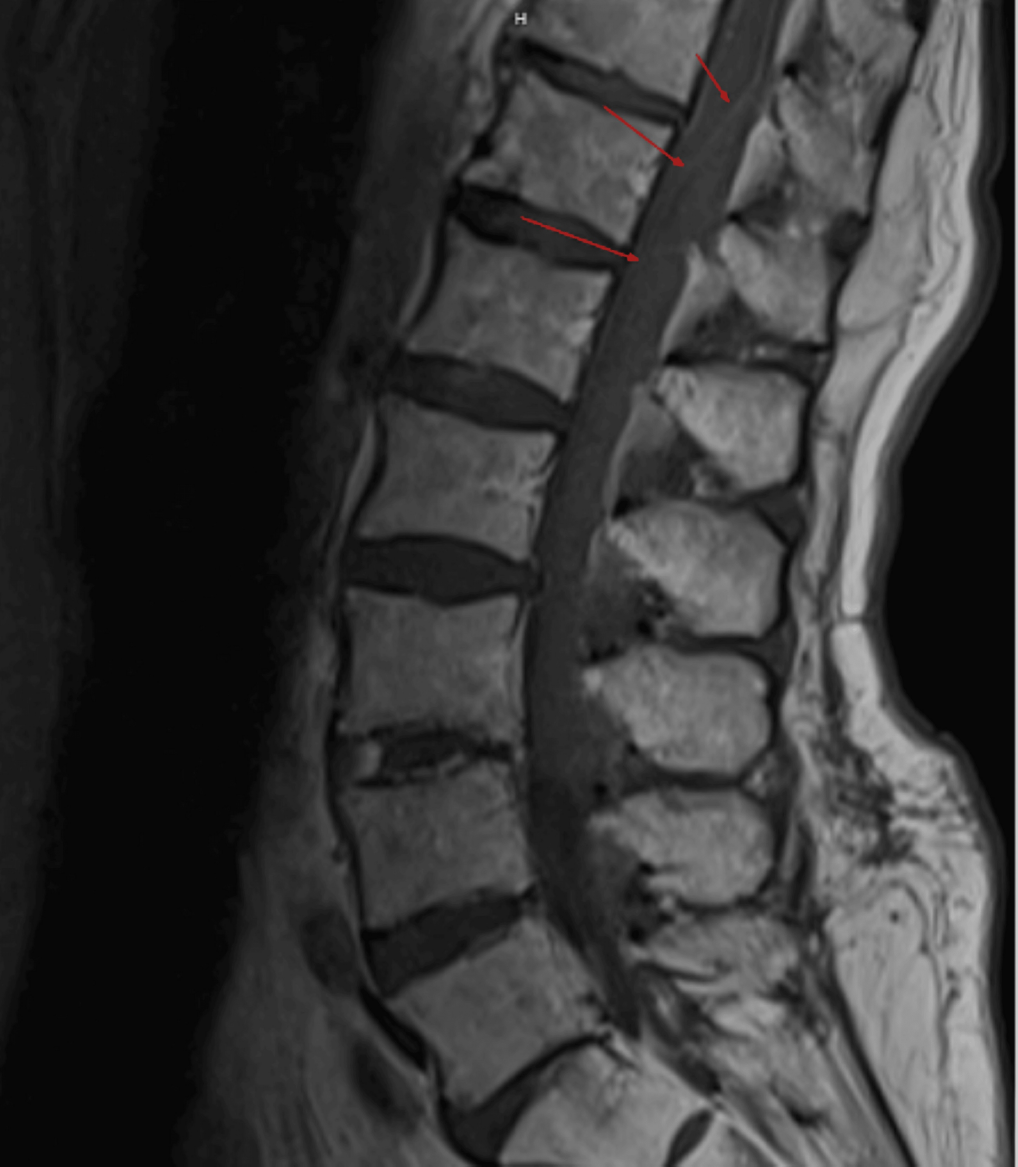
T1 post-contrast sagittal MRI. There is no enhancement of the delineated hyperintense mass within the spinal canal. MRI: magnetic resonance imaging

**Figure 5:**
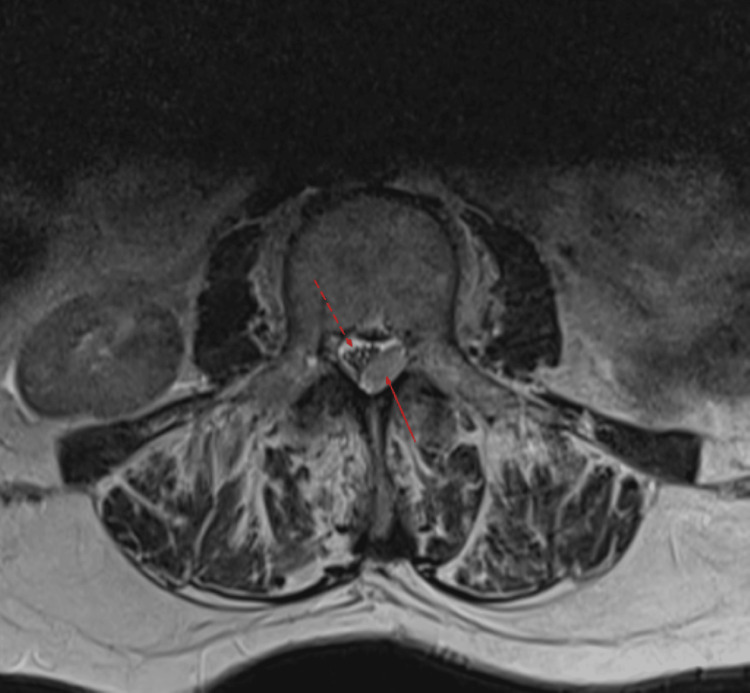
T2 axial MRI demonstrates mass within the spinal canal (solid arrow) displacing the cord anterolaterally (dashed arrow). MRI: magnetic resonance imaging

On the inpatient floor, an MRI of the lumbar spine was obtained that evening and verbally reported the following morning. Imaging revealed an epidural hematoma extending from T11 to L4 (Figures 1-5). A neurosurgeon was consulted, and the patient underwent emergency surgery for hematoma extraction and decompressive laminectomy of T11-L3. After the operation, she could move her lower limbs with a greater range of motion, and her muscle strength was approximately 4/5. The pain over the lower limbs was relieved, and the only pain she experienced was over the surgical site. Pain was controlled with acetaminophen and as-needed tramadol for breakthrough pain. The patient was discharged to a subacute rehab facility to regain further strength and function.

## Discussion

This case underscores the importance of considering SEH in patients lacking conventional risk factors, such as a history of trauma. In this case, the patient could ambulate to the restroom approximately three hours prior to presentation in the emergency department representing an acute change from baseline. Initial laboratory and imaging workup did not reveal an immediate cause for the patient's acute leg weakness, and with a lack of recent trauma and noncausal findings on CT imaging, emergent findings were presumed to be ruled out. While the initial workup was unrevealing, the acuity of the presentation merits consideration of SEH, especially given her history of long-term use of clopidogrel, age, and symptoms suggestive of a lumbar cord injury. 

Given the infrequency of this condition, clinicians may not be familiar with the spectrum of clinical presentations for SEH. A retrospective case-control study published in 2022 involved the largest SEH sample size to date, over 1,100 patients [5]. In this study, 79% of the patients presented with sudden-onset neck or back pain. There was preponderance for males with a male-to-female ratio of 1.5:1. Involvement of specific spinal cord segment displayed a bimodal distribution; an age younger than 50 years correlated with cervicothoracic involvement, while an age older than 50 years correlated with thoracolumbar involvement [4]. In our case, the patient indeed presented with acute lower back pain and was 86 years old with a thoracolumbar hematoma. These characteristics provide valuable insight into when to consider the diagnosis of SEH. 

In addition to these clinical features, one may consider anticoagulation or antiplatelet therapy as a risk factor and prognostic factor. A literature review of 1,000 cases of SEH found 48 cases in which antiplatelet therapy has been reported as the sole etiologic factor without coagulopathy [2]. Furthermore, some case reports have suggested that clopidogrel was a significant factor in the development of SEH [6-11]. While these studies suggest antiplatelet use is relatively common in patients with SEH, a recent meta-analysis of 617 patients concluded that antiplatelet use is comparable to the general population and thus may not be a risk factor for developing SEH [3]. In this meta-analysis, anticoagulation was found in a much higher proportion than the public. The authors concluded that anticoagulation was a risk factor for developing SEH. Additionally, this study assessed surgical outcomes using multivariate regression. Not surprisingly, the results demonstrated an association with good surgical outcomes and patients not on anticoagulation or antiplatelet therapy. Additionally, the use of antiplatelet therapy was not conclusively associated with better surgical outcomes compared to the use of vitamin K inhibitors [3]. Regarding anticoagulation, long-term use has been associated with worse outcomes, increased probability of recurrence, and larger hematomas [3,5,12]. While the evidence is less conclusive regarding antiplatelet therapy and further study is merited, we recommend holding anticoagulation and antiplatelet medications at the time of diagnosis and considering these as risk factors for SEH. 

Well-established prognostic factors are the preoperative neurological deficit and the time from symptom onset to intervention [13-16]. Neurological deficits have been quantified in the SEH literature by different scales including the Frankel scale and the American Spinal Injury Association impairment scale [3,12,15]. Regardless of the scale used, significant weakness or paresthesias is an indication for surgical decompression [14]. Some literature reports improvement with conservative therapy in patients with minor pain and paresthesias or patients who had improvement in their symptoms [6,17]. However, the standard treatment is rapid diagnosis and surgical management of severe symptoms or mild symptoms that do not show signs of early improvement [15]. Most literature suggests an optimal operative interval of 12-24 hours [3,5,8,13,18,19]. Importantly, the operative interval begins with symptom onset rather than admission to the hospital [5]. 

## Conclusions

Taken together, our patient was at high risk for a poor outcome given her preoperative neurological dysfunction and extended operative interval. However, the patient underwent successful decompression and hematoma extraction within 32 hours and resolution of symptoms in the following 24 hours. While SEH is uncommon, it is a potentially reversible cause of paralysis if diagnosed and treated emergently. If initial imaging and laboratory workup are unremarkable, the patient should ideally undergo an emergent MRI. Although conservative management may be acceptable in rare cases, surgical intervention should be performed as soon as possible, ideally within 12 hours of symptom onset.
